# Buffalo as a Field for Physicians

**Published:** 1871-01

**Authors:** 


### Editorial.

### Buffalo as a Field for Physicians.

We have received the following letter, (one of many,) which we will answer through the pages of the *Journal*, hoping that a reply to this one will be regarded as satisfactory reply to many of similar nature:

"——— Jan. 15, 1871.

"Dr. J. F. Miner:

"My Dear Sir,—I hope you will indulge me, while I ask of you a word of advice. I graduated, as you know, in medicine, last spring, class of 1870, and after spending a few weeks at home, I commenced looking for a suitable place to commence practice. Thus far I have been unsuccessful, and now would like to change for any favorable field you may know of, where I can obtain a footing. Buffalo, or the vicinity would suit me best; and I would like to know how the matter stands in the city, or in any of the larger neighboring towns. My former acquaintance with you, and your uniform readiness to oblige me, emboldens me to ask a statement of conditions, with which your extensive acquaintance and careful observation must make you familiar. I shall, therefore, wait anxiously your reply; and shall be guided by your advice.

" Yours, most Truly,

" M. C. C——."

"Buffalo, Jan. 15th, 1871.

" My Dear Doctor:

" Your very polite and confidential note reached me in due time, and I hasten to make a brief reply, to a young friend, whose real interests I would gladly promote.

" Buffalo offers a wide field for medical practice; though, so called physicians are at every corner: most of them constantly seeking for employment in their professions; and, I am sorry to add, without any very signal success. You have spoken of my observation in these matters, and so I am inclined to give you its results, in connection with the general statement of facts, and you are at liberty to use these conclusions as may suit you best.

" I believe that a young physician of good ability, and thorough education, *can* seek for medical practice in a manner to be richly rewarded—can do so in Buffalo, or any other city or town, where his inclinations may lead him. My observation convinces me, that the general rule of failure is owing to the man and not to the place. I have never known a "*political*" doctor to achieve success in his profession; and I suppose the principal reasons are sufficiently obvious. Neither have I ever known a "*religious*" doctor—that is, a doctor religions in his associations for unworthy and sinister motives—who ever attain any success as a physician. I have never known the physician of a society, party or clique, who depended upon such influences, to find any ample field for honorable practice. The ground in Buffalo, and every where else, is fully occupied with competitors in this manner of seeking for medical business.

" Still, I think, we have a favorable field in this vicinity, and that, if you are as good a physician as you used to be student, you need have no hesitation what course to pursuse. You are young now, and in the very prime of life; you can do whatever your ambition and industry may dictate. You seem inclined to make Buffalo your home; and, if you conclude to do so, I can assure you of a warm and hearty welcome on the part of the members of the profession. But, perhaps, it will not be quite fair, if I do not speak a word as to what is necessary to attain anything which can, in true sense, be called success.

You are not coming to compete with political, sectarian or otherwise employed physicians, who, as I said, are stationed at every corner—these have very little which you desire. But you are to compete with men who spend all their efforts in professional pursuits; who observe disease, or study its nature continually—interrupted only when worn nature insists upon repose—which is often broken in upon by the urgent solicitations of the unfortunate, who will not allow them rest. They have no amusements, few social opportunities. They give up all time to, and have no pleasure except in, professional pursuits. These men, who have already enjoyed considerable experience, are yet *students*: they generally work about twelve hours in the twenty-four, visiting and prescribing for the sick; and they *study* medicine often, the remaining hours.

"There is no obvious "opening," though the number is not great, for they are men, sick people can always find, and always find them ready to answer to their calls; they have a clear eye, a steady hand, a firm and manly purpose; and to compete with them, you must possess equal energy, activity, education and industry. Now, my dear doctor, if you think that you can give up every thing else, and study and practice medicine continuously the remainder of your natural life, forgetting all other pursuits and devoting yourself to it alone, then Buffalo is a favorable field—you will be "the right man in the right place."

"Yours very truly, &c.,

"J. F. MINER."

